# Global hotspots in the present-day distribution of ancient animal and plant lineages

**DOI:** 10.1038/srep15457

**Published:** 2015-10-26

**Authors:** Şerban Procheş, Syd Ramdhani, Sandun J. Perera, Jason R. Ali, Sanjay Gairola

**Affiliations:** 1 Discipline of Geography, University of KwaZulu-Natal, Westville Campus, P.B. X54001, Durban 4000, South Africa.; 2 School of Life Sciences, University of KwaZulu-Natal, Westville Campus, P.B. X54001, Durban 4000, South Africa; 3 Department of Natural Resources, Sabaragamuwa University, P.O. Box 2, Belihuloya 70140, Sri Lanka; 4 Department of Earth Sciences, University of Hong Kong, Pokfulam Road, Hong Kong, China

## Abstract

The current distribution of biotic lineages that emerged in the deep time has both theoretical and practical implications, in particular for understanding the processes that have forged present-day biodiversity and informing local and regional-scale conservation efforts. To date however, there has been no examination of such patterns globally across taxa and geological time. Here we map the diversity of selected extant seed plant and tetrapod vertebrate lineages that were already in existence either in the latest Triassic or latest Cretaceous. For Triassic-age linages, we find concentrations in several regions – both tropical and temperate – parts of North America, Europe, East and South-east Asia, northern South America, and New Zealand. With Cretaceous-age lineages, high values are relatively uniformly distributed across the tropics, with peak the values along the Andes, in South-east Asia and Queensland, but also in the temperate Cape Mountains. These patterns result from a combination of factors, including land area, geographic isolation, climate stability and mass extinction survival ability. While the need to protect many of these lineages has been long recognised, a spatially-explicit approach is critical for understanding and maintaining the factors responsible for their persistence, and this will need to be taken forward across finer scales.

Earth’s present-day biotic inventory represents the outcome of four billion years of interactions between numerous processes and phenomena involving the solid planet, the oceans and atmosphere, catastrophic volcanism, bolides and life itself. The origins of the extant forms can be traced back to different times in the geological past, some being very deep, others much nearer – but each traceable to a specific ancestral lineage at any instant in the past. An important associated question concerns the distribution of ancient lineages across the planet. Are they spread evenly around the globe or is there evidence for clustering? If there are anomalous regions, or even “hotspots”, why is this so? Furthermore, from a conservation perspective identified regions of high ancient lineage diversity, some of which may not have been previously recognized, would present obvious value^1^,^2^.

Today, global patterns in the diversity of life are no longer measured strictly as numbers of species; species relatedness is also viewed as critical^1^,^2^,^3^,^4^,^5^,^6^,^7^. The increasing availability of dated phylogenies for various plant and animal groups means that measures such as phylogenetic diversity^1^,^8^ (PD) and phylogenetic endemism^2^ (PE) can now be exploited, and mapped regionally as well as globally. Exciting patterns are emerging, some indicating similarities between species-based and phylogenetic measures, others highlighting interesting differences.

With regards to terrestrial vertebrates, recent global studies have focused on mammals^3^, birds^4^, and amphibians^5^, as a consequence of their detailed distribution data. Unfortunately, far less is known about plants. At the regional scale some detailed PD maps have been produced (e.g. South Africa’s Cape Floral Kingdom^1^ and Australia^6^). Beyond that, the closest thing to a plant PD map is an estimate of higher-taxon diversity^7^. Consequently, there are no PD studies collating or comparing plants and animals.

When evaluating where ancient lineages survive and their relative representation across biotas, there are also methodological issues. Maps of PD fail to account for the difference between a few ancient lineages and a multitude of recent ones. One way to circumvent this involves the mapping of lineage diversity for narrow time windows in the past^3^. The survival of ancient lineages may have been facilitated in regions that have been (a) climatically-stable over long periods^9^, (b) protected from the impact of mass extinction drivers^10^, (c) more isolated^11^, and therefore protected from the invasion of novel predators or competitors^12^, or d) large enough to ensure survival stochastically – some part of the region remaining suitable for a lineage at any given point in time, as consistent with species-area relationships^13^. Here we map globally the diversity of large and representative plant and vertebrate lineages of ages^14^,^15^,^16^,^17^,^18^ that immediately pre-date the last two mass extinctions (end-Triassic, ~201 Mya, and end-Cretaceous, ~66 Mya), and discuss how the observed patterns tally with the various explanations outlined above.

## Results

Ten seed plant and eighteen tetrapod vertebrate lineages have survived since the Late Triassic, most of them classified at family level or higher (see Supplementary Information for details). The two groups are dominated respectively by gymnosperms and amphibians, with nine and eleven lineages each. Chelonians are limited to two, while the ancestors of extant angiosperms, rhynchocephalians, squamates, crocodylians, birds and mammals are each represented by one. For the latest Cretaceous, 21 angiosperm lineages from the order Poales are recovered, almost all at family level, with the grass family (Poaceae) represented by four. The subclass Campanulidae is represented by 34, mostly at family or subfamily level; the daisies (Asteraceae) form a single lineage. Among the tetrapods, the birds are recovered as 39 lineages at or above family level (passerines representing just two of these), and the mammals as 41, mostly above family level (one monotreme, three marsupials, and the rest placentals) (see Supplementary Information).

**Figure 1:**
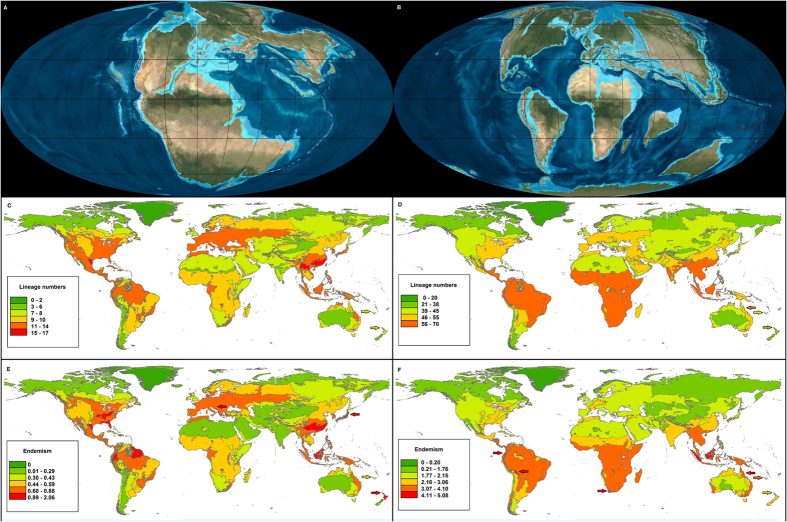
Continental arrangement at the time of the last two mass extinction events, and the diversity of extant lineages of Triassic and Cretaceous ages. (**A,B**), Continental arrangement at the Triassic-Jurassic (**A**) and Cretaceous-Paleogene (**B**) boundaries (Copyright Ron Blakey Colorado Plateau Geosystems, Inc.). (**C–F**) The present-day diversity of ancient lineages as compiled in the present study, based on dated phylogenies and distribution data (see Methods section) and mapped in ArcGIS ver. 9.3^39^. Lineages of Late Triassic (**C,E**) and Late Cretaceous (**D,F**) age – untransformed (**C,D**) and weighted to indicate where narrowly-distributed lineages are more numerous (**E,F**).

The diversity of Cretaceous lineages paints a mostly typical latitudinal gradient, though it is notable (and arguably counterintuitive) to see that rainforest is not in itself unusually diverse. With regards to the end-Triassic lineages, rainforest stands out from surrounding non-rainforest ecoregions, but it is also notable that several temperate regions have high values, and overall the latitudinal gradient is less pronounced than is the case for the end-Cretaceous forms (Fig. 1; Supplementary Information).

When transforming the data to highlight endemism, unusually high end-Triassic values are mostly associated with the former Laurasian continents: parts of North America (mostly based on the narrowly distributed amphibian lineages), as well as Europe and East Asia (where multiple salamander and gymnosperm lineages overlap), with an extension in northern South America, where recently colonising salamanders overlap with several Neotropical lineages, some narrowly distributed. Other top values are in Borneo and Palawan, where the endemic *Barbourula* frogs, from a lineage otherwise absent in the tropics, overlap with a broad assortment of other tetrapods and gymnosperms, and northern New Zealand, where *Leiopelma* frogs and the only living rhynchocephalian, *Sphenodon*, survive (Extended Data Fig. 1). For the latest Cretaceous, by contrast, high values are in the former Gondwana continents (mostly South America for birds and Poales, Africa for mammals, and Australasia for Campanulidae; Extended Data Fig. 2) – the few overall outlying values are for some insular South-east Asian and Andean foothill ecoregions, for South Africa’s Mountain Fynbos ecoregion, and for the Queensland rainforests (Fig. 1).

It is worth noting that there is little overlap between the Triassic- and Cretaceous- lineage hotspots, the only ones being insular South-east Asia, and adjacent ecoregions in northern South America. Nevertheless, high (if not exceptionally high) values are also recorded in both cases through much of the Neotropics, in both tropical and montane parts of Africa, southern China, Indochina, New Guinea, and eastern Australia. Endemic or nearly endemic groups in at least one age category are also found in Chile, southern Africa’s Cape, Madagascar, northern New Zealand and New Caledonia, although overall impoverishment due to insularity or climatic isolation means that these regions do not necessarily stand out in both cases (some, in neither) in terms of overall endemism. These areas stand out more clearly on the endemism maps compared to the lineage diversity maps (Fig. 1).

## Discussion

Although there are geographical differences between the various groups included (Extended Data Fig. 1 and 2), our combined results (Fig. 1) are likely to be robust, and the highest diversity regions are also noted for hosting ancient lineages in groups not included here^19^. The distribution patterns identified for the extant taxa are also confirmed by the latest known occurrences in extinct ones. Animal and plant groups that were already distinct at the end-Triassic, but were only lost after the end-Cretaceous mass extinction include the Bennettitales^20^, Albanerpetonidae^21^, and possibly the Meiolaniidae^22^. Their occurrences confirm the refugial function of both the large northern landmasses and the lesser southern continents and islands (e.g., Australia, New Caledonia).

Multiple processes are compressed in the patterns of present day diversity for ancient lineages. Survival of the extinction event relevant to their recorded age was in some cases followed by range expansion and subsequent secondary contraction. In order to persist to the present and feature on the maps, Triassic lineages had to survive not only the end-Triassic extinction event, but the end-Cretaceous one too. Lineages of both ages recorded here also had to endure aridification and cooling in the Cenozoic, including the particularly taxing Quaternary ice-house. Without doubt, the sharp latitudinal differences illustrated here in Cretaceous lineages would have been less pronounced prior to the onset of Quaternary glacials and interglacials. Due to this, the maps summarizing untransformed lineage numbers, while informative for global conservation efforts, are limited in explaining the survival of ancient plant and animal groups. On the other hand, the maps of transformed values (endemism in Fig. 1 – see Methods) highlight regions rich in range-restricted lineages. Under a null scenario of survival in the areas of current occupancy, these are the areas with higher long-term refugial value. This interpretation provides some support for each of the four hypotheses tested in this paper.

At the end of the Triassic, the continents were largely contiguous as parts of Pangea (Fig. 1), so in this case differential survival is not necessarily an indication that parts of the globe were less affected – plants and animals would have still been able to recolonize fairly easily those regions where they had become extinct. Clearly changes in non-marine biotic assemblages during the end-Triassic mass extinction are documented from North America (pollen/spores assemblages) and South Africa (vertebrates)^23^. Unfortunately, this is too little to deduce whether the extinctions exhibited geographic patterns. While the causes for this mass extinction remain contentious, most of the proposed mechanisms would likely involve global effects^1^,^24^,^25^.

This suggests that the enhanced survival of Triassic-age lineages in the Northern Hemisphere (Fig. 1) is a mass effect – with large areas of mesic to humid environments allowing for the survival of both gymnosperms and amphibians. For most of the Triassic, the majority of world’s regions were not particularly humid, and at a global scale precipitation was likely strongly seasonal (monsoon-dominated^25^). However, the occurrence of extensive humid episodes subsequently forced most Triassic-age lineages to adapt to a humid world, and these only endured subsequent Cenozoic aridification in those areas where humidity persisted.

Southern Hemisphere survival in the Cretaceous-age groups may relate to increased isolation following the breakup of Pangea (~200 Mya) and later Gondwana (~165 Mya). Isolated areas are less likely to be affected by the biotic turmoil following mass extinctions^12^,^26^, but the biota involved may remain cases of “survival without recovery”^12^. Southern landmasses (South America, Africa, New Guinea) also harbour some of the largest areas of tropical rainforest. As shown by our maps (Fig. 1), tropical rainforest is not outstanding in its levels of ancient lineage preservation, but may have had a certain mass effect in the case of Cretaceous lineages, in the face of mid-Cenozoic climate aridification^27^.

It can be argued that the precise timing of radiations in the groups considered here is not yet certain. Conflicting values to this effect can be found as regards, e.g., mammal radiation^14^,^28^. The bulk of their diversification was presented in OneZoom^14^ as pre-dating the end-Cretaceous extinction, which made us include mammals as one of our study groups. Conversely, the monocotyledon order Poales (which includes grasses, sedges and rushes), is considered here as a second hyperdiverse and dominant plant group with lineages of a similar age as the Campanulidae^14^. However, the ages of the Poales families are much younger in OneZoom^17^. Nevertheless, the current distributions of lineages in these groups are relevant to mass extinction survival patterns, irrespective of the precise timing of their diversification. Post-extinction radiations in some groups could have been caused by extinctions in others, niches left vacant were filled by new colonisers, while yet other groups survived in areas less affected by this turmoil due to geographical isolation^12^. Thus, the distributions of lineages approximating the age of mass extinction events are indicative of survival of either mass extinction as such, or at the very least of the turbulent periods that followed it.

The conservation implications of our study are two-fold. First, it is of great concern that some of the areas where most ancient lineages are present, East and South-east Asia, are located where habitat transformation is currently extreme^29^. Second, when trying to preserve narrowly-distributed ancient linages, we contend that tropical rainforests should not necessarily be considered priority targets. More important are several isolated Southern Hemisphere hotspots, including the rainforests of Queensland, New Zealand, and the Cape Region of South Africa, as well as parts of the Northern Hemisphere.

New Zealand is particularly interesting in that it hosts relictual lineages^19^ from both age windows, although these have not necessarily survived there ever since the mass extinction events discussed, but more likely arrived there in the Cenozoic via overwater dispersal^30^. Thus in some cases the islands are repositories of Antarctic or Australian biotas that were subsequently extirpated on the once host continents as they respectively became fully glaciated, or too arid. At the same time, though, other New Zealand plants and animals have been lost in large numbers since the end of the Cretaceous^31^. Notably, the two oldest lineages restricted to New Zealand, *Sphenodon* and *Leiopelma*, were brought close to this ending after human occupation, and today survive best on offshore islands. This is a stark reminder that the relictual function of such regions can easily be disrupted through human activities.

## Methods

In order to account for the effects of mass extinction events on lineage distribution, we focused on lineages with extant representatives that were present 201 Mya and 66 Mya (thus having survived the end-Triassic and end-Cretaceous extinction events, respectively). Initially, all tetrapod vertebrate and seed plant lineages present 201 Mya were listed. For those present 66 Mya, we focused the analysis on the two most diverse and ecologically dominant lineages in either group, the birds and mammals for tetrapods, and the Campanulidae (also known as Asteridae II, inclusive of the daisy family, Asteraceae) and Poales (inclusive of grasses, Poaceae) among seed plants^32^. Lineage divergence dates for each group were derived from the most comprehensively sampled phylogenies available, unless these were incompatible with the age confidence intervals derived from better calibrated, dedicated phylogenetic studies for narrower groups. Thus, initially, lineage lists were compiled based on node age values provided online from the OneZoom website ( http://www.onezoom.org/)^17^, which is a comprehensive source of information on the phylogenetic relationships of both tetrapod vertebrates and seed plants. These were found to be largely compatible with age values provided in dedicated phylogenies for tetrapods, birds, mammals, and Campanulidae, despite recent debates, especially in the case of mammals. In the case of spermatophytes, some lineages are presented in OneZoom as older than in dedicated studies^14^,^15^,^16^; and in Poales, divergence dates from OneZoom are substantially younger than in published dedicated phylogenies^14^,^33^,^34^, which were consequently followed.

Lineage distributions were then mapped at ecoregion level. In the case of animals, distribution data were derived from the WildFinder website ( http:// worldwildlife.org/science/wildfinder/), as previously processed^35^, with additional data for recently discovered taxa (Chikilidae^36^, *Laonastes*^37^ and *Karsenia*^38^). For plants these were digitised using the Angiosperm Phylogeny website ( http://www.mobot.org/MOBOT/research/APweb/) and the Conifers.org website ( http://conifer.org/), with additional information extracted from Flora Malesiana ( http://floramalesiana.org/), Flora of New Zealand ( http://floraseries.landcareresearch.co.nz/pages/Index.aspx), and Flora of China ( http://www.efloras.org/flora_page.aspx? flora_id=2).

While the definition and delimitation of global WildFinder ecoregions are often imprecise across finer scales, these represent a useful set of geographic units for testing global hypotheses, and the fairly coarse grain made digitising plant lineage distributions a realistic task.

The diversity of extant lineages of Triassic and Cretaceous age was mapped in ArcGIS ver. 9.3.^39^ untransformed, as well as corrected for the total area of occupancy of each lineage, to highlight ecoregions where narrowly distributed ancient lineages occur. In this latter case, presences were summed after being divided by the square root of the number of ecoregions occupied, thus essentially representing a measure of endemism^40^. In each case, the values were presented using a colour scale from green to red to represent ecoregion-level values from the bottom whiskers, through the bottom and top sections of the box and top whiskers to top outliers^35^. Bottom outliers typically represented small oceanic islands, and are omitted in the colour legends.

## Additional Information

**How to cite this article**: Procheş, Ş. *et al.* Global hotspots in the present-day distribution of ancient animal and plant lineages. *Sci. Rep.* **5**, 15457; doi: 10.1038/srep15457 (2015).

## Supplementary Material

Supplementary Figures

Supplementary Table S1
