# Supplementary material for: Global hotspots in the present-day distribution of ancient animal and plant lineages
Source: Sci Rep. 2015 Oct 26;5:15457. doi: 10.1038/srep15457 (PMC4620499; doi:10.1038/srep15457)
Supplement: Supplementary Figures [file srep15457-s1.doc]

**Global hotspots in the present-day distribution of ancient animal and plant lineages**

Şerban Procheş, Syd Ramdhani, Sandun J. Perera, Jason R. Ali & Sanjay Gairola

Extended Data


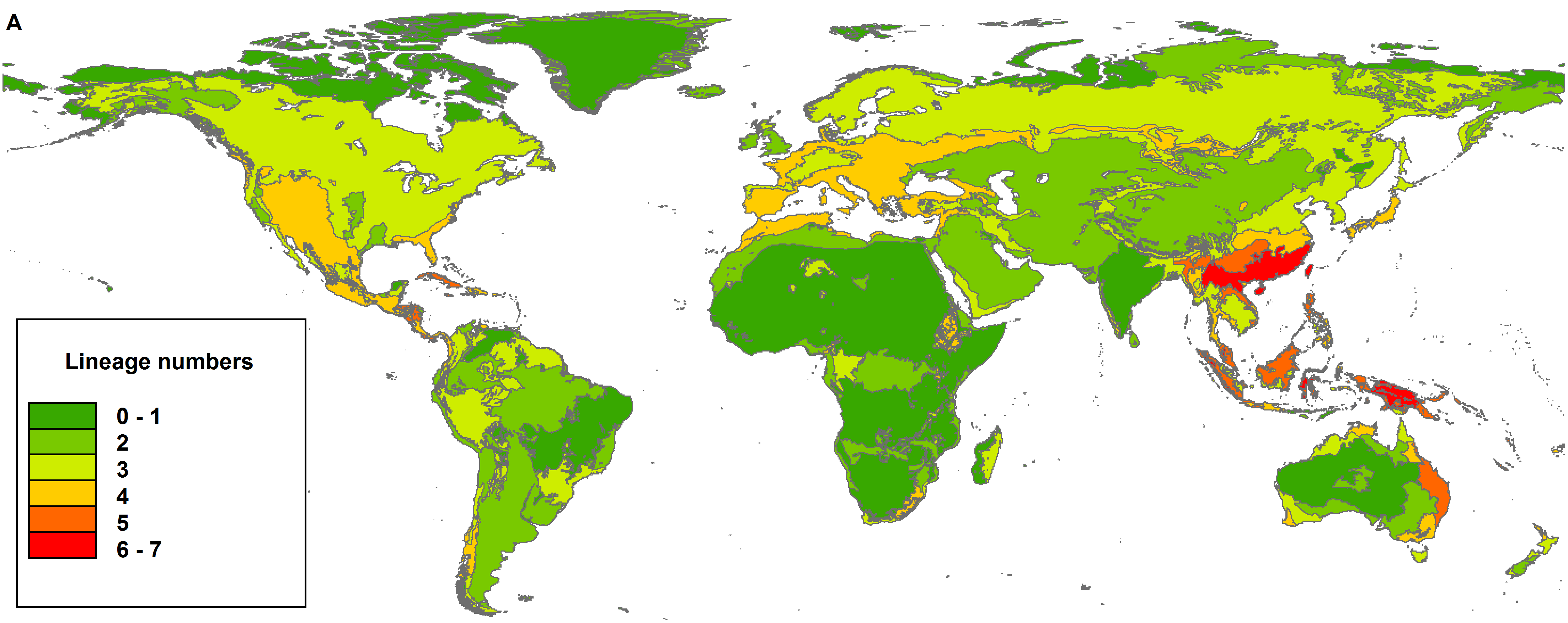


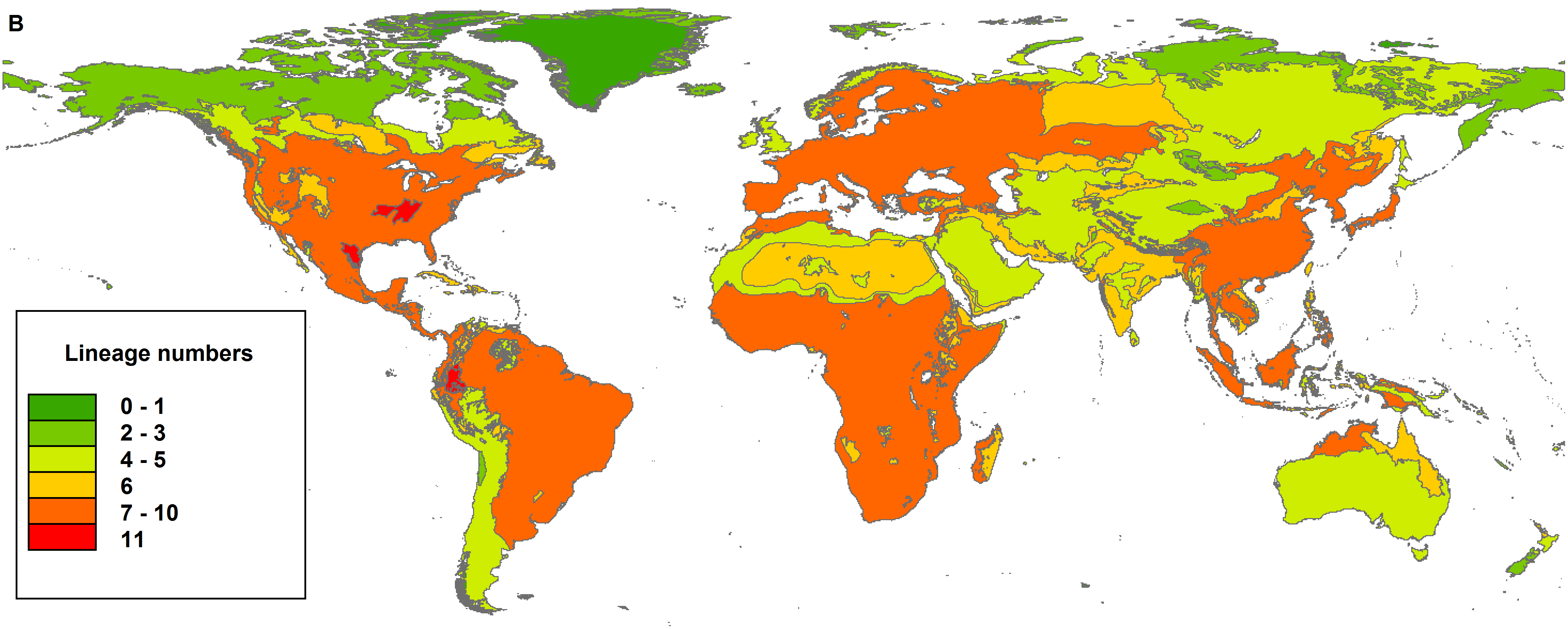


**Extended Data Figure 1** | **Maps of diversity (untransformed number of lineages) in Triassic-age lineages. a,** Spermatophyta. **b,** Tetrapoda. Compiled in the present study, based on dated phylogenies and distribution data (see Methods section) and mapped in ArcGIS ver. 9.3.


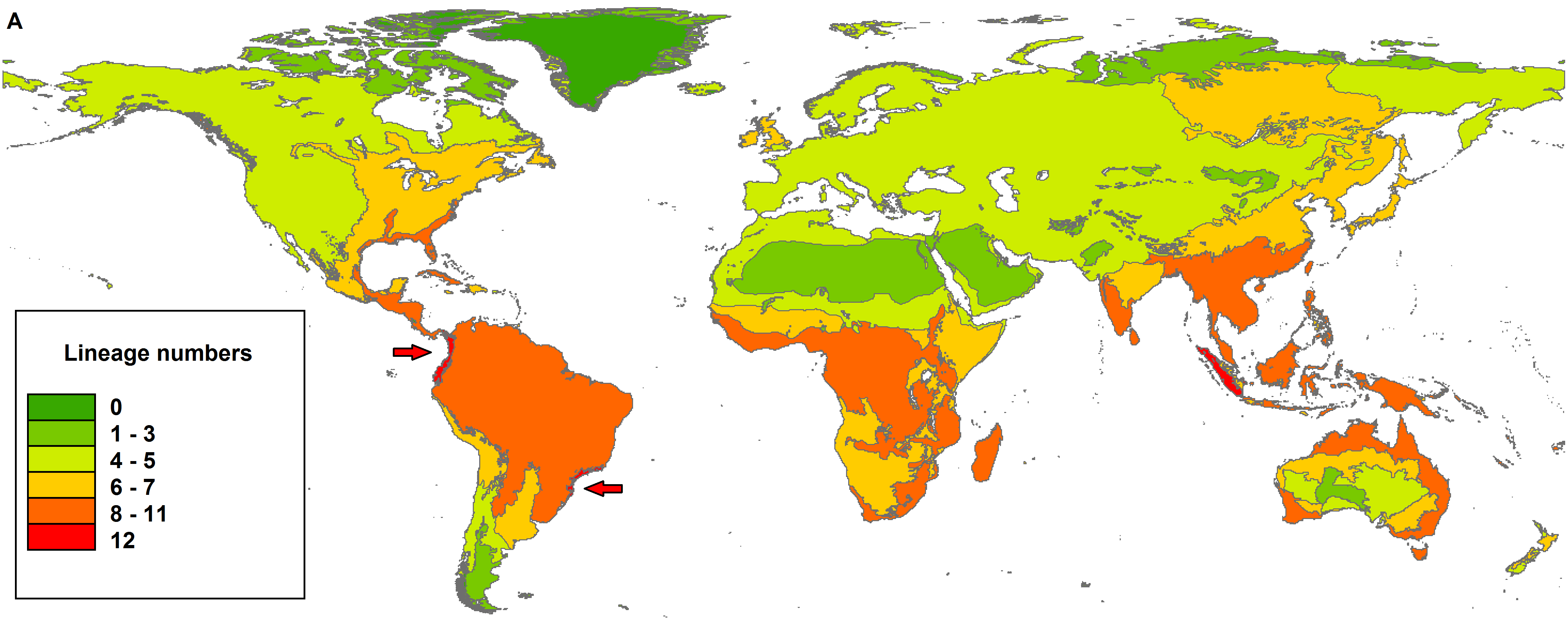


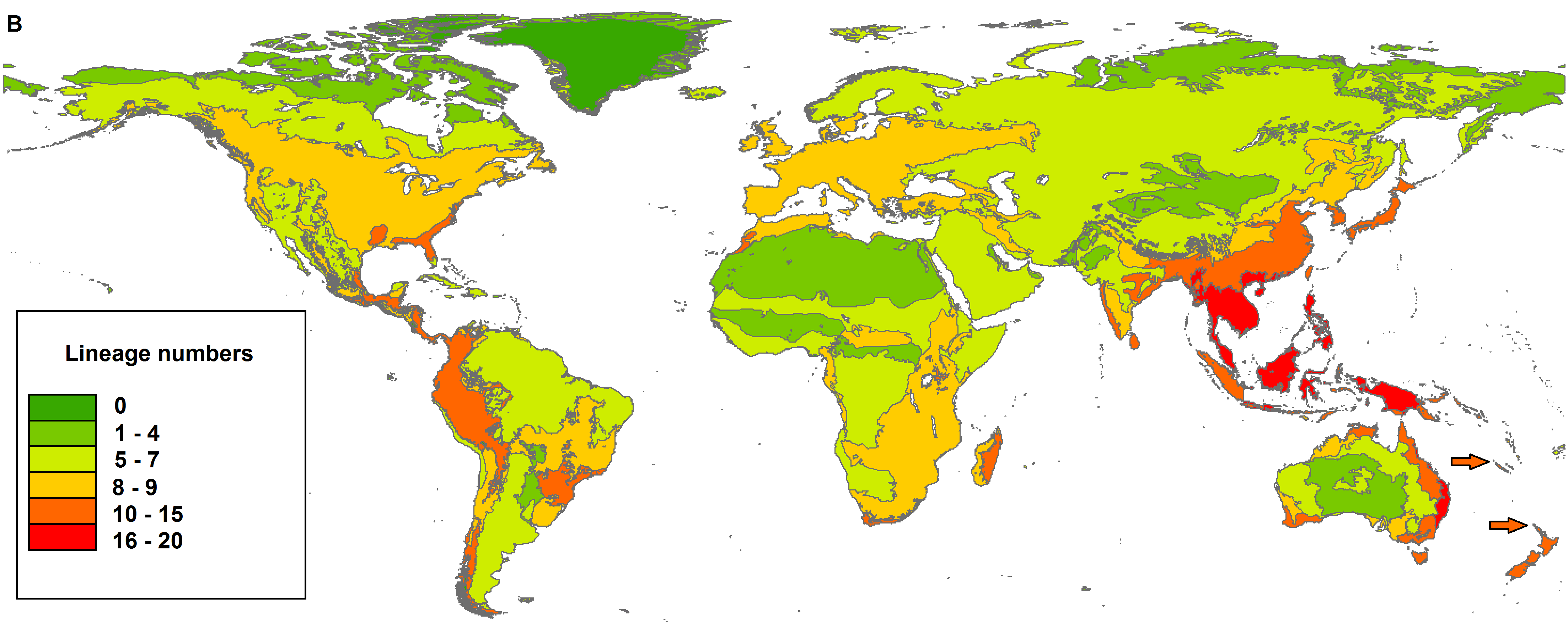


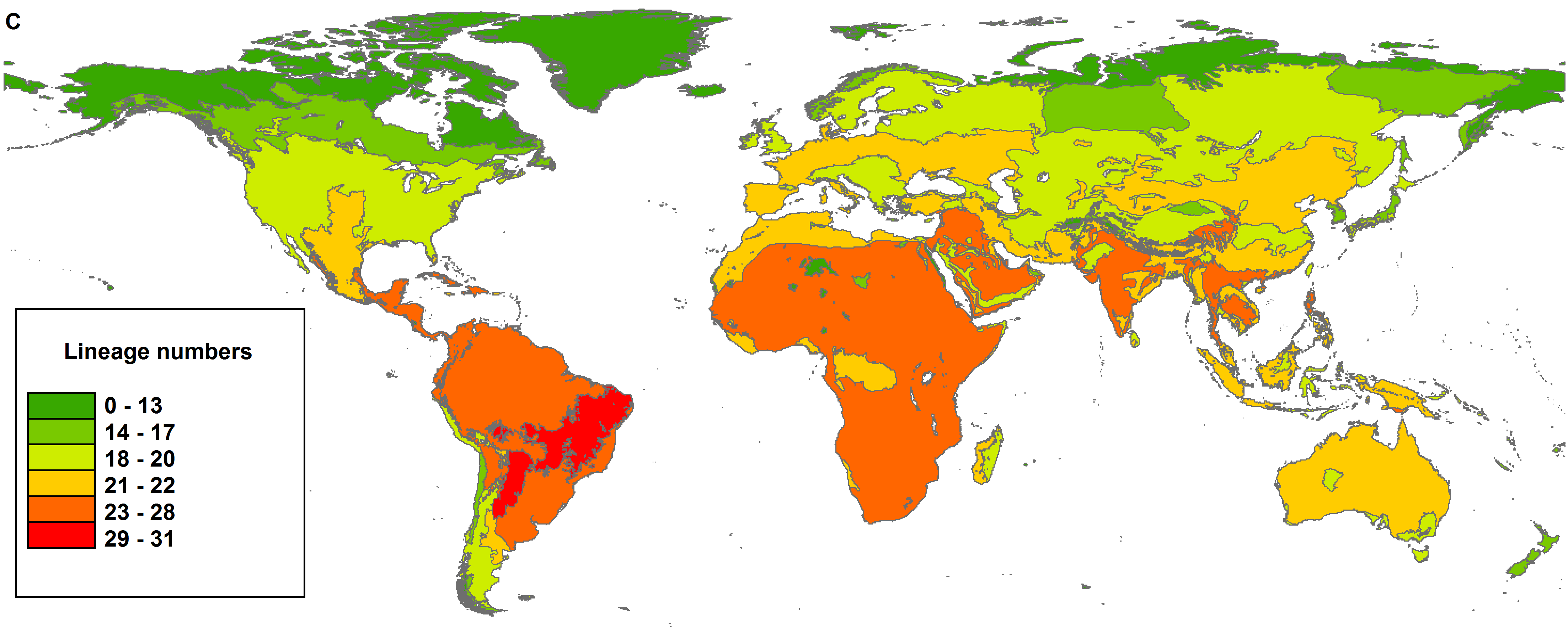


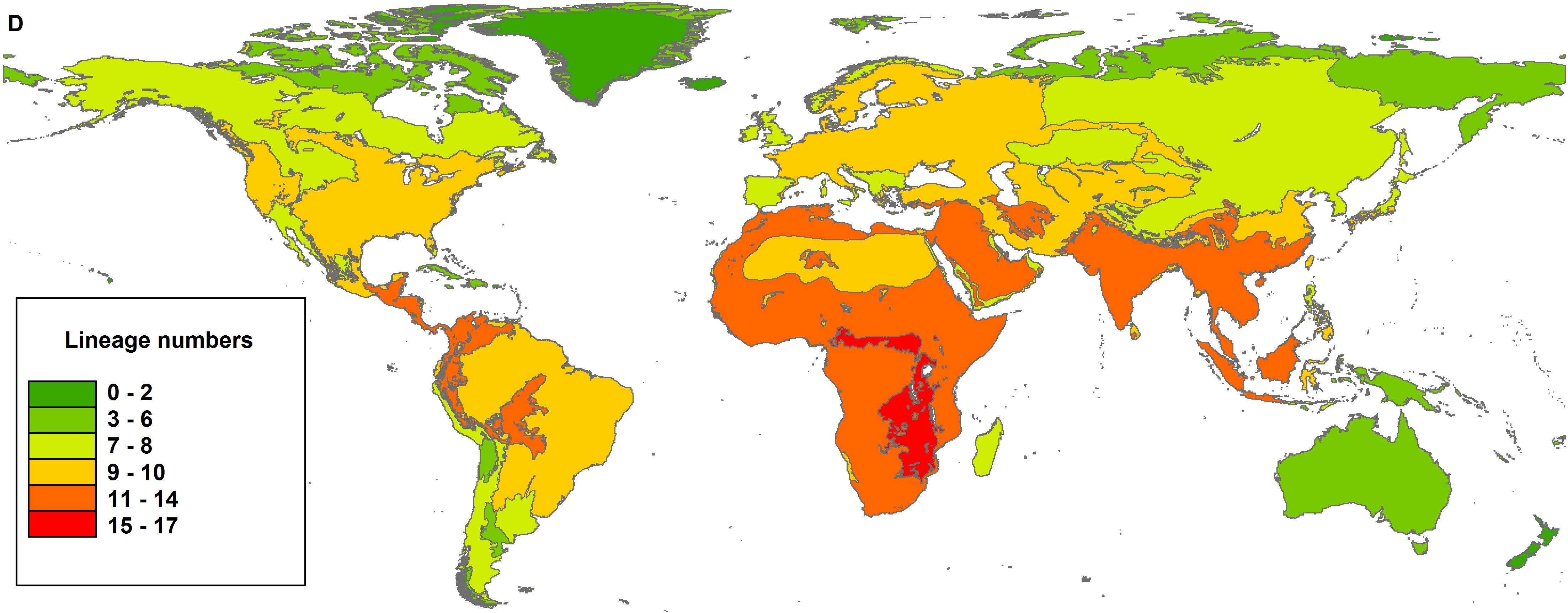


**Extended Data Figure 2** | **Maps of diversity (untransformed number of lineages) in Cretaceous-age lineages. a,** Poales. **b**, Campanulidae. **c,** Aves. **d,** Mammalia. Compiled in the present study, based on dated phylogenies and distribution data (see Methods section) and mapped in ArcGIS ver. 9.3.
